# Platelet‐Rich Fibrin and Its Derivatives as Adjuncts to Dental Implant Osseointegration: A Scoping Review

**DOI:** 10.1155/ijod/6146734

**Published:** 2026-09-16

**Authors:** P. Gitanjali, Neetha J. Shetty, Amitha Basheer, Shruti Singh, Nandini Nagpal, Shreya Hegde

**Affiliations:** ^1^ Department of Periodontology, Manipal College of Dental Sciences Mangalore, Manipal Academy of Higher Education, Manipal, Karnataka, India, manipal.edu; ^2^ Department of Public Health Dentistry, Manipal College of Dental Sciences Mangalore, Manipal Academy of Higher Education, Manipal, Karnataka, India, manipal.edu; ^3^ Department of Conservative dentistry and Endodontics, Manipal College of Dental Sciences Mangalore, Manipal Academy of Higher Education, Manipal, Karnataka, India, manipal.edu

**Keywords:** A-PRF, dental implants, implant stability, injectable PRF, L-PRF, osseointegration, platelet-rich fibrin, resonance frequency analysis

## Abstract

**Background:**

Platelet‐rich fibrin (PRF) is a family of autologous platelet concentrates whose solid and liquid formulations differ in cellular distribution, fibrin architecture, handling, and intended clinical use. Contemporary evidence suggests a possible influence on early peri‐implant healing, but long‐term osseointegration and implant‐survival advantages are uncertain.

**Objective:**

To map human clinical evidence on leukocyte‐ and PRF (L‐PRF), advanced PRF (A‐PRF), injectable or liquid PRF (i‐PRF/liquid PRF), and related PRF formulations used as adjuncts to dental implant osseointegration, with attention to preparation protocols, outcome methods, healing phase, and clinical limitations.

**Methods:**

A scoping review was conducted in accordance with JBI guidance and PRISMA‐ScR. PubMed/MEDLINE, Web of Science Core Collection, and Embase were searched for English‐language human clinical studies published from 2015 to 2025. Eligible studies assessed a PRF formulation during implant placement or site preparation and reported implant stability, radiographic bone outcomes, histological bone formation, or peri‐implant clinical outcomes. Evidence was synthesized by PRF formulation, outcome modality, and early (within 3 months) versus later (6 months or longer) observation.

**Results:**

Nineteen human clinical studies met the eligibility criteria. RFA/ISQ was the most common outcome and frequently suggested a transient improvement in secondary stability during the first weeks of healing. Several radiographic studies reported favorable bone‐density or marginal bone findings, whereas others found no between‐group differences. Histological evidence was sparse and was not interchangeable with RFA or radiographic surrogate outcomes. Studies with follow‐up of 6–12 months did not establish consistent superiority in marginal bone preservation, implant survival, or other long‐term clinical endpoints. Preparation reporting was incomplete: rpm and time were often stated, but relative centrifugal force (RCF), rotor geometry, tube composition, and device specifications were commonly missing.

**Conclusions:**

PRF and its derivatives show promising potential as adjuncts that may accelerate early healing and secondary implant stability. The available evidence does not establish durable superiority for osseointegration or implant survival, nor does it identify one formulation as clinically superior. Standardized randomized trials with complete preparation reporting, validated outcome measures, long‐term follow‐up, patient‐reported outcomes, and economic evaluation are required.

**Summary**

Platelet‐rich fibrin is prepared from a patient’s own blood and can be applied as a membrane, clot, or liquid during implant treatment. Across the clinical studies mapped in this review, PRF most often appeared to help during the first weeks of healing, when the bone is forming around an implant and mechanical stability is changing to biological stability. However, the studies used different PRF preparations and different measurements, and the longer studies did not consistently show that PRF produces better bone levels or implant survival. PRF should therefore be described as a promising optional adjunct rather than a proven routine requirement. The studies also did not adequately measure pain, satisfaction, quality of life, or cost from the patient’s perspective.

## 1. Introduction

Osseointegration refers to the direct structural and functional linkage between the viable bone and the surface of a load‐bearing implant. The process commences with the development of clots and inflammatory signals, succeeded by angiogenesis, recruitment of osteoprogenitors, deposition of woven bone, and subsequent remodeling. Initial stability during insertion is primarily mechanical, but subsequent stability arises physiologically as new bone develops and matures at the interface of the adjunctive implant. Biologic methods should be assessed in relation to the healing stage at which an outcome is evaluated.

Autologous platelet concentrates have transitioned from anticoagulant‐reliant platelet‐rich plasma (PRP) formulations to fibrin‐based solutions created without external anticoagulants. Recent analyses differentiate between first‐ and second‐generation concentrates and highlight that the name PRF does not refer to a singular standardized substance [1–3]. The ultimate outcome is contingent upon the blood collection tube, the interval from venipuncture to centrifugation, the centrifugal force, rotor configuration, duration of spinning, processing apparatus, and postcentrifugation management.

Traditional leukocyte‐ and platelet‐rich fibrin (L‐PRF) creates a robust clot or membrane comprised of a fibrin matrix, platelets, leukocytes, and signaling proteins. Advanced PRF (A‐PRF) employs reduced centrifugal conditions to modify the cellular composition within the clot. Injectable or liquid PRF (i‐PRF) employs a brief, low‐pressure technique to preserve a temporary liquid state that can either cover implant surfaces or be combined with particle grafts [4–7]. Recent alterations encompass concentrated PRF (C‐PRF), aimed at enhancing platelet and leukocyte output, alongside horizontal‐centrifugation techniques devised to mitigate the cell‐gradient impacts of fixed‐angle rotors [8–12]. These items must not be regarded as physiologically or clinically identical.

The biological justification for PRF is credible: its fibrin matrix serves as a temporary scaffold and can discharge mediators linked to platelets and leukocytes that are integral to angiogenesis, cellular migration, proliferation, and extracellular matrix development. Nonetheless, biological plausibility and in vitro cellular activity do not confirm clinical osseointegration. Additives and surfaces of tubes can influence the product; silica‐coated plastic tubes have sparked worries regarding microparticle pollution and cytotoxicity, highlighting the necessity to disclose tube composition and system attributes [13–15].

Clinical investigations have evaluated PRF by resonance frequency analysis (RFA), insertion torque, radiographic bone levels or density, clinical peri‐implant metrics, and occasionally histological examination. These techniques address many inquiries. RFA measures the rigidity of the implant‐bone assembly instead of the direct contact between bone and implant; radiographic images offer dimensional or gray‐scale proxies but fail to directly assess microscopic integration; histological analysis can examine tissue‐level contact but typically necessitates a biopsy or retrieval method. Integrating these results without methodological differentiation may exaggerate the confidence. Prior evaluations indicate that PRF could enhance secondary implant stability in the first healing phase, although the extent and duration of this advantage remain ambiguous [16, 17]. The current scoping review thus assesses PRF as a comprehensive category of formulations and centers clinical synthesis on the osseointegration of dental implants. Soft‐tissue results are preserved as secondary supportive outcomes because of their limited number, which does not warrant a more expansive designation.

The review question was as follows: In humans receiving dental implants, what evidence exists regarding the effects, preparation methods, and clinical limitations of PRF formulations (including L‐PRF, A‐PRF, and i‐PRF/liquid PRF) used as adjuncts to implant osseointegration, and how do findings differ between early healing and later clinical follow‐up?

## 2. Methods

### 2.1. Design and Protocol

This scoping review was reported with the PRISMA Extension for Scoping Reviews (PRISMA‐ScRs) and followed updated JBI methodological guidance [18, 19]. The protocol is available on the Open Science Framework (DOI: 10.17605/OSF.IO/R537E). The review was designed to map the types of PRF intervention, clinical applications, outcome methods, and gaps in the heterogeneous literature rather than to estimate a single pooled treatment effect.

The protocol‐defined concept encompassed the PRF family, including L‐PRF, A‐PRF, i‐PRF/liquid PRF, T‐PRF, and concentrated growth factors (CGF). The review question and synthesis therefore addressed PRF formulations collectively while preserving the formulation‐specific interpretation of the findings.

### 2.2. Study Design and Eligibility Criteria

This scoping review was conducted in alignment with the PRISMA‐ScR 2018 guidelines.

#### 2.2.1. Population (P)

Human participants undergoing dental implant placement, including immediate or delayed implant procedures, were included, with or without adjunctive regenerative interventions.

#### 2.2.2. Concept (C)

Clinical use of PRF in implant therapy, including all available formulations (e.g., L‐PRF, A‐PRF, i‐PRF, T‐PRF, and CGF), and its impact on implant stability, osseointegration, peri‐implant‐bone changes, and soft‐tissue healing.

#### 2.2.3. Context (C)

Clinical implant dentistry settings across all geographic regions were studied.

#### 2.2.4. Inclusion Criteria

1. Research was conducted on humans receiving dental implants, immediate or delayed.
2. Research using any form of PRF in dental implants which include:
  • L‐PRF
  • A‐PRF
  • i‐PRF
  • T‐PRF
  • CGF
  • PRF as a standalone material or PRF combined with grafting material
3. Outcomes associated with the following:
  • Dental implant stability (ISQ/RFA)
  • Osseointegration of dental implants
  • Marginal bone changes around implants
  • Bone density changes around implants
  • Healing of peri‐implant tissues
  • Changes in width of keratinized mucosa
  • Peri‐implant parameters clinically
4. Primarily, research methodologies, which include the following:
  • Randomized controlled trials
  • Nonrandomized clinical trials
  • Prospective and retrospective observational study designs
  • Comparative clinical studies
  • Case reports and case series
5. Studies conducted within the year January 2015 through December 2025.
6. Publications that were written in English.
7. Peer‐reviewed articles with full‐text availability.

#### 2.2.5. Exclusion Criteria

Studies were excluded if they met any of the following criteria:

1. Nonhuman studies, including animal experiments and in vitro laboratory investigations.
2. Finite element analysis or purely experimental biomechanical studies without clinical application are used.
3. Studies evaluating platelet concentrates other than PRF (e.g., PRP) without the inclusion of PRF as an intervention.
4. Narrative reviews, editorials, expert opinions, letters to the editor, and conference abstracts are included without primary clinical data.
5. Studies not related to dental implant therapy (e.g., PRF used solely for periodontal surgery without implant placement).
6. Articles not published in English or without an accessible full text.

### 2.3. Information Sources and Search Strategy

Three electronic databases were searched: PubMed/MEDLINE, the Web of Science Core Collection, and Embase. The searches covered records published from January 2015 to December 2025. Reference lists of relevant reviews and included reports were checked to identify potentially eligible records. Database syntax was adapted to each platform, and the core concepts are shown in Table 1.

**Table 1:** Core database search strategy.

PubMed:	((“dental implants”[Title/Abstract] OR “dental implants”[MeSH Terms] OR “dental implants”[MeSH Terms] OR “dental prosthesis”[MeSH Terms]) AND “loattrfull text”[Filter] AND ((“dental prosthesis”[Title/Abstract] OR “dental prosthesis”[MeSH Terms] OR “dental prosthesis”[MeSH Terms] OR “dental prosthesis”[MeSH Terms]) AND “loattrfull text”[Filter]) AND ((“platelet rich fibrin”[Title/Abstract] OR “platelet rich fibrin”[MeSH Terms] OR “platelet rich fibrin”[MeSH Terms] OR “platelet rich fibrin”[MeSH Terms] OR “platelet rich fibrin”[MeSH Terms] OR “platelet rich fibrin”[MeSH Terms]) AND “loattrfull text”[Filter])) AND ((fft[Filter]) AND (2015:2026[pdat]))
Web of science:	Dental Implant (All Fields) and platelet rich fibrin (All Fields)
Embase	(“dental implant”/exp OR “dental implant” OR ((“dental”/exp OR dental) AND (“implant”/exp OR implant))) AND (“platelet‐rich fibrin”/exp OR “platelet‐rich fibrin”)

### 2.4. Selection and Data Charting

After deduplication, titles and abstracts were screened, followed by a full‐text eligibility assessment. Data were charted for author/year, design, sample, implant context, PRF subtype and application, comparator, blood volume and tube, rpm and/or relative centrifugal force (RCF), centrifugation time, centrifuge/rotor, outcome modality, follow‐up, and principal findings. Missing technical details were recorded as not reported (NR) rather than inferred. Because RCF depends on rpm and rotor radius, RCF was not back‐calculated when the rotor radius was unavailable.

### 2.5. Critical Appraisal and Synthesis

A formal risk‐of‐bias or certainty‐of‐evidence assessment was not undertaken. This decision reflects the scoping purpose of mapping a heterogeneous evidence base and is consistent with JBI guidance, which does not require critical appraisal for every scoping review [19]. Consequently, this review does not rank studies by methodological quality or make causal claims. Instead, a structured methodological appraisal was incorporated into the synthesis by considering study design, sample size, comparator, follow‐up, PRF protocol completeness, and the biological meaning and limitations of each outcome modality.

For interpretive clarity, outcomes measured within 3 months were categorized as early healing because this interval commonly spans the transition from mechanical primary stability to biological secondary stability. Outcomes reported at 6 months or longer were categorized as a later clinical follow‐up. This pragmatic grouping was used for narrative synthesis and does not imply a universal biological threshold.

## 3. Results

### 3.1. Study Selection

**Figure 1:**
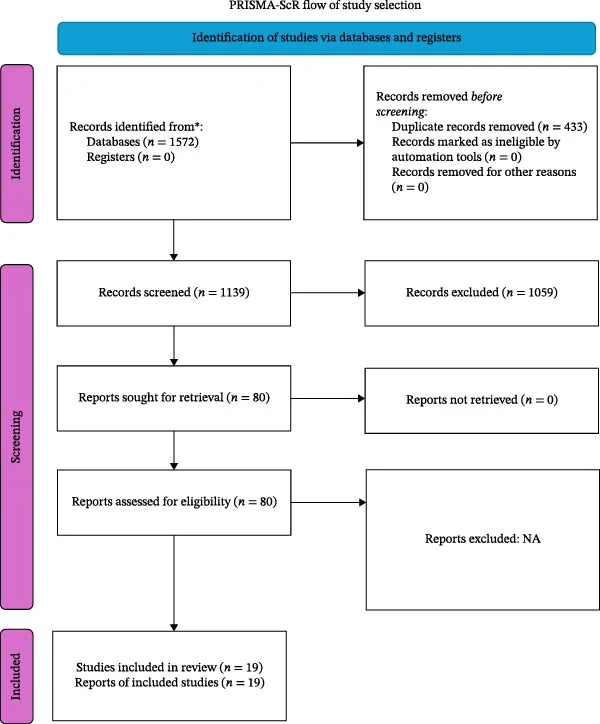
PRISMA flow diagram showing the study selection process for the scoping review.

The database searches identified 1572 records. After the removal of 433 duplicates, 1139 records underwent title and abstract screening, of which 1059 were excluded. Eighty full‐text reports were assessed for eligibility, and 61 were excluded. Nineteen human clinical studies met the inclusion criteria and were incorporated into the evidence map (Figure 1).

### 3.2. Study Characteristics and PRF Formulations

The 19 studies included randomized parallel‐group trials, split‐mouth randomized trials, prospective comparative clinical studies, and one case series. L‐PRF or an unspecified solid PRF was the most common. A smaller group evaluated A‐PRF for socket preservation or i‐PRF/liquid PRF for implant‐surface coating or mixing with a xenograft. No eligible trial directly compared L‐PRF, A‐PRF, and i‐PRF under otherwise identical conditions; therefore, no formulation can be judged clinically superior. Study characteristics and outcome‐phase interpretations are presented in Table 2, and preparation protocols in Table 3.

**Table 2:** Included human clinical studies: design, outcomes, follow‐up, and phase‐specific interpretation.

Author and year	Study design	Aim of study	Sample size	Type of PRF used	Comparator/control	Outcome measured	Key findings	Conclusion
Kapoor et al. 2022 [20]	Split‐mouth RCT	Evaluate effect of PRF on implant stability (osseointegration)	21 patients, 60 sites	PRF	Implant without PRF	Implant stability (ISQ via RFA)	Higher ISQ at 1 week and 1 month in PRF group; no difference at 3 months	PRF improves early osseointegration
Mohye et al. 2022 [21]	Randomized clinical trial	Assess i‐PRF+graft in filling implant gap and tissue outcomes	16 patients	i‐PRF (injectable PRF)	Bone graft alone	Bone density, crestal bone level, and gingival thickness	Bone density significantly higher with i‐PRF; no major difference in bone height	i‐PRF improves bone quality but limited effect on dimensions
Öncü and Alaaddinoğlu 2015 [22]	Within‐patient randomized clinical study; 20 patients; ≥2 adjacent implants each	To evaluate PRF’s effect on early implant stability.	20 patients; 64 implants (PRF: 31, control: 33)	Conventional L‐PRF membrane; 2700 rpm for 12 min	Implant placement without PRF	ISQ by RFA at baseline, 1 and 4 weeks	Significantly higher ISQ with PRF at 1 week and 4 weeks	PRF improved early implant stability and may accelerate osseointegration.
Ali et al. 2025 [23]	Split‐mouth RCT	Assess clinical and radiographic effects of PRF on osseointegration	21 patients, 60 implants	PRF	No PRF	Bone density, marginal bone loss, probing depth, and healing index	Higher bone density, less bone loss, and better healing in PRF group	PRF enhances osseointegration and peri‐implant healing
Hasanzade et al. 2025 [24]	Clinical study (split‐mouth)	Evaluate i‐PRF effect on implant stability	20 patients (40 implants)	i‐PRF	No i‐PRF	Implant stability (ISQ)	Significant increase in stability in i‐PRF group	i‐PRF improves implant stability during healing
Awasthi et al. 2025 [25]	Clinical comparative study	Evaluate implant mobility, bleeding, and bone level with/without PRF in esthetic zone	40 patients	PRF	Immediate implant without PRF	Implant mobility, bleeding on probing, and marginal bone level	PRF group showed lower mobility, less bleeding, and better bone levels	PRF improves clinical and radiographic outcomes in immediate implants
Sharma et al. 2023 [26]	Randomized clinical study	Assess soft and hard tissue changes with PRF around implants	15 patients	PRF	No PRF	Crestal bone loss, probing depth, and soft‐tissue indices	Less bone loss and better soft‐tissue healing in PRF group	PRF enhances peri‐implant tissue stability
Lima et al. 2022 [27]	Case series	Evaluate PRF membranes for mucosal thickness augmentation	8 patients	PRF membranes	No control	Mucosal thickness and ridge defect	Significant increase in mucosal thickness and ridge improvement	PRF useful for soft‐tissue augmentation
Boora et al. 2015 [28]	Randomized controlled trial	Evaluate PRF effect on peri‐implant soft tissue and crestal bone	20 patients	PRF	No PRF	Bone level, probing depth, and bleeding	Less crestal bone loss in PRF group; soft‐tissue similar	PRF beneficial for bone preservation
Naeimi Darestani et al. 2023 [29]	Randomized clinical trial	Assess effect of L‐PRF on implant stability and bone loss	~30 patients	L‐PRF	No PRF	Implant stability (ISQ) and marginal bone loss	No significant difference between groups	L‐PRF shows no added benefit in stability or bone loss
Anapu et al. 2024 [30]	Clinical study	Evaluate implant stability with PRF using RFA	13 patients (26 implants)	PRF	No PRF	Primary and secondary stability (ISQ)	Higher secondary stability in PRF group	PRF enhances osseointegration over time
Tamilarasan et al. 2024 [31]	Clinical study	Evaluate primary and secondary stability with PRF	24 patients	PRF	No PRF	ISQ, PI, GI, SBI, and IPPD	Higher implant stability at 3 and 6 months; lower probing depth	PRF improves implant stability and peri‐implant health
Chary et al. 2021 [32]	RCT	Evaluate bone quality and torque with A‐PRF at 6 vs. 8 weeks	20 patients	A‐PRF	6‐week vs. 8‐week placement	Bone formation and insertion torque	Higher bone formation and torque at 8 weeks	A‐PRF improves early implant success timing
Tabrizi et al. 2018 [33]	Split‐mouth RCT	Evaluate implant stability in posterior maxilla	20 patients	PRF	No PRF	ISQ at 2, 4, and 6 weeks	Significantly higher stability in PRF group at all time points	PRF improves early implant stability
de Oliveira Fernandes et al. 2022 [34]	Split‐mouth pilot study	Evaluate i‐PRF coating on implants	10 patients	i‐PRF	Standard implants	ISQ stability	No significant difference between groups	i‐PRF comparable to advanced implant surfaces
Jalaluddin et al. 2025 [35]	In vivo clinical study	Evaluate implant stability and marginal bone level with/without PRF	40 patients	PRF	Implant without PRF	Implant stability (ISQ), marginal bone level	Significantly higher implant stability and better bone preservation in PRF group at 6 months	PRF enhances osseointegration and improves implant stability during early healing
Soni et al. 2023 [36]	Randomized controlled trial; single‐blinded	To evaluate the clinical and radiographic efficacy of L‐PRF membrane in immediate postextraction implant placement	18 immediate‐implant sites; 9 control and 9 test sites	Leukocyte–platelet‐rich fibrin membrane	Immediate implant placement without L‐PRF membrane	Peri‐implant probing depth, tissue biotype, implant stability, marginal bone loss, and peri‐implant radiolucency	L‐PRF showed marginal improvement in tissue biotype and slightly reduced peri‐implant probing depth and marginal bone loss at 6 months, but differences were statistically nonsignificant	L‐PRF may provide some benefit in improving soft‐tissue thickness and healing around immediate implants, but due to small sample size and short follow‐up, stronger evidence from larger studies is needed
Güvenç et al. 2022 [37]	Clinical comparative study	To investigate the effect of injectable platelet‐rich fibrin on implant stability	40 implants placed in 15 patients aged 25–67 years with mandibular edentulous areas	Injectable platelet‐rich fibrin—i‐PRF	Implants placed without i‐PRF	Implant stability measured by resonance frequency analysis; ISQ values recorded at surgery, 1st, 2nd, and 4th weeks	Control group showed a statistically significant decrease in mean ISQ values during the 1st week. The i‐PRF group showed increasing ISQ values, with statistically significant increases at the 2nd and 4th weeks	i‐PRF positively influenced early implant stability and may promote peri‐implant‐bone healing; it can be safely used as an adjunct in dental implant surgery
Diana et al. 2018 [38]	Randomized, single‐blind, and controlled clinical trial	To evaluate whether PRF improves clinical and radiographic outcomes of immediate implants	41 implants in 31 subjects; final analysis: 39 implant sites in 29 subjects	Autologous platelet‐rich fibrin	Immediate implants without PRF augmentation	Implant stability quotient, clinical and radiographic outcomes	Implant stability increased significantly in both groups over 3 months, but no significant difference was found between PRF and control groups	PRF did not significantly improve osseointegration or implant stability in immediate implants with adequate primary stability

*Note:* Study design labels reflect reporting in the source articles; they are not risk‐of‐bias ratings.

Abbreviations: CBCT, cone‐beam computed tomography; i‐PRF, injectable platelet‐rich fibrin; ISQ, implant stability quotient; L‐PRF, leukocyte‐ and platelet‐rich fibrin; mo, month(s); RCT, randomized controlled trial; RFA, resonance frequency analysis; wk, week(s); yr, year.

**Table 3:** PRF preparation and processing variables reported by the included studies.

Study	Subtype/use	Blood and tube	Speed/RCF	Time	Centrifuge/rotor	Reporting appraisal
Kapoor et al. 2022 [20]	Solid PRF; osteotomy/implant site	Tube/volume NR	3000 rpm; RCF NR	10 min	IntraSpin; rotor geometry NR	rpm and time reported; tube, radius and RCF absent
Mohye et al. 2022 [21]	i‐PRF mixed with xenograft	Additive‐free plastic/hydrophobic tube; volume NR	700 rpm; ~60 ×*g* reported	3 min	Model/rotor NR	Low‐speed liquid protocol reported; device detail incomplete
Öncü and Alaaddinoğlu 2015 [22]	L‐PRF in socket; liquid fraction on implant	Tube/volume NR	2700 rpm; RCF NR	12 min	Model/rotor NR	Conventional protocol reported incompletely
Ali et al. 2025 [23]	PRF at implant placement	NR	NR	NR	NR	Preparation variables not sufficiently reported for reproducibility
Hasanzade et al. 2025 [24]	i‐PRF at implant site	10 mL; white vacuum tube	700 rpm; RCF NR	3 min	EBA 20 S, Hettich; rotor geometry NR	Volume, tube description, model, and rpm/time reported; RCF/radius absent
Awasthi et al. 2025 [25]	Solid PRF; immediate implant	NR	3000 rpm; RCF NR	10 min	Model/rotor NR	Rpm/time reported; most technical variables NR
Sharma et al. 2023 [26]	Solid PRF around implant	10 mL; sterile anticoagulant‐free Vacutainer	3000 rpm; RCF NR	10 min	Digital centrifuge; model/rotor NR	Tube class and protocol reported; RCF/device geometry absent
Lima et al. 2022 [27]	PRF membranes for mucosal augmentation	NR	NR	NR	NR	Insufficient preparation reporting in accessible report
Boora et al. 2015 [28]	Solid PRF at one‐stage placement	Volume/tube NR	3200 rpm; RCF NR	12 min	R‐8C REMI; rotor geometry NR	Model/rpm/time reported; tube and RCF absent
Naeimi Darestani et al. 2023 [29]	L‐PRF at posterior‐maxillary site	2 × 10 mL; glass‐coated plastic tubes	2700 rpm; RCF NR	12 min	IntraSpin; rotor geometry NR	Tube, model, and rpm/time reported; RCF/radius absent
Anapu et al. 2024 [30]	Solid PRF with implant placement	Volume/tube NR	3000 rpm; clot RCF reported as ~400 ×*g*	12 min	REMI; swing‐out head reported	Relatively complete, but tube and rotor radius absent
Tamilarasan et al. 2024 [31]	Solid PRF at implant site	10 mL; tube type NR	3000 rpm; RCF NR	12 min	Model NR; PRF box (GDC) for compression	Volume/rpm/time and handling reported; tube/device/RCF incomplete
Chary et al. 2021 [32]	A‐PRF plugs/membrane for socket preservation	30 mL in 3 A‐PRF tubes	1500 rpm; RCF NR	14 min	Model/rotor NR	Subtype, volume, and rpm/time reported; device/RCF absent
Tabrizi et al. 2018 [33]	Solid PRF in implant site	Tube/volume NR	2800 rpm; RCF NR	12 min	IntraSpin; rotor geometry NR	Model/rpm/time reported; tube and RCF absent
de Oliveira Fernandes et al. 2022 [34]	Liquid PRF coating implant surface	Tube/volume NR	2000 rpm; 268 ×*g* reported	10 min	Model/rotor NR	Rpm and RCF reported; tube/device details incomplete
Jalaluddin et al. 2025 [35]	Solid PRF during implant placement	NR	NR	NR	NR	Preparation variables not sufficiently reported for reproducibility
Soni et al. 2023 [36]	L‐PRF membrane; immediate implant	10 mL; VACUTECH PRF tubes	2700 rpm; RCF NR	12 min	Dentist’s centrifuge, labtech; manual mode	Tube/model/rpm/time reported; RCF and rotor geometry absent
Güvenç et al. 2022 [37]	i‐PRF at implant placement	Tube/volume NR	700 rpm; RCF NR	3 min	Process for PRF system; rotor geometry NR	Model/rpm/time reported; tube/RCF absent
Diana et al. 2018 [38]	Solid PRF; immediate implant	NR	NR	NR	NR	Preparation variables insufficiently reported

*Note:* NR, not reported in the source article or accessible study report used for data charting. rpm values were not converted to RCF (relative centrifugal force) when rotor radius was unavailable. Approximate RCF values are retained only where reported by the study or its protocol. Differences in tube composition, time to spin, rotor radius/angle, and clot compression can alter the final PRF product even when rpm and duration appear similar.

### 3.3. Early Healing Outcomes (Within 3 Months)

RFA/ISQ was the dominant early outcome. Kapoor et al. [20], Öncü and Alaaddinoğlu [22], Tabrizi et al. [33], Anapu et al. [30], Güvenç et al. [37], Hasanzade et al. [24], and other clinical investigations reported higher or more rapidly recovering ISQ values in PRF‐treated sites during the first weeks or months. These findings are compatible with an accelerated transition to secondary stability. However, primary stability at insertion was not consistently different, which is biologically expected because it is largely determined by bone quality, implant geometry, osteotomy preparation, and insertion mechanics rather than by a freshly applied biologic concentrate.

The early signal was not uniform. Diana et al. [38] found increasing stability in both PRF and control groups without a significant between‐group difference at 3 months. de Oliveira Fernandes et al. 34] similarly did not demonstrate a clear stability advantage from liquid‐PRF implant coating despite follow‐up to 1 year. Thus, a higher early ISQ in some trials should be interpreted as a possible acceleration of healing, not proof of greater final bone‐to‐implant contact.

Histology was uncommon. Chary et al. [32] harvested bone cores after A‐PRF socket preservation and reported more bone formation and higher insertion torque at 8 weeks than at 6 weeks. Because both groups received A‐PRF and differed in healing time, this study supports time‐dependent maturation after A‐PRF‐treated socket preservation but does not isolate the treatment effect of A‐PRF against a no‐PRF control.

### 3.4. Later Clinical Outcomes (6 Months or Longer)

Later stability findings were less consistent. Mohye et al. [21] reported higher radiographic bone density at 6 months when i‐PRF was mixed with bovine bone mineral, but no clear improvement in gingival biotype and no direct evidence that i‐PRF alone changed dimensional bone outcomes. Several studies reported favorable marginal bone or clinical indices at 6–12 months [23, 25, 26, 28, 31, 35], whereas Naeimi Darestani et al. [29], Soni et al. [36], Diana et al. [38], and De Oliveira Fernandes et al. [34] reported no significant or only marginal between‐group differences for important bone or stability outcomes.

Only limited evidence extended to 12 months, and none of the included studies was designed or powered to establish a durable difference in implant survival. Consequently, the mapped literature supports—at most—a possible early‐healing advantage. It does not establish the long‐term clinical superiority of PRF over conventional implant placement.

### 3.5. Outcome‐Method Appraisal

Clinical indices such as probing depth, bleeding, plaque, healing scores, and mucosal thickness describe the peri‐implant soft‐tissue environment but do not directly measure osseointegration. RFA/ISQ is noninvasive and repeatable, yet it reflects stiffness of the implant‐bone complex and is influenced by implant design, position, transducer orientation, cortical engagement, and surrounding bone; it is not a direct measurement of bone‐to‐implant contact [39, 40]. Insertion torque is a placement‐time mechanical measure and cannot be treated as secondary biological stability.

Standardized periapical radiography is suitable for mesiodistal marginal bone level change but cannot resolve buccolingual dimensions. CBCT provides three‐dimensional anatomy, although gray values are device‐ and acquisition‐dependent and generally cannot be assumed to equal calibrated Hounsfield units [41, 42]. Histomorphometry provides the most direct tissue‐level evidence of new bone or bone‐to‐implant contact but is rare in the human evidence and may require invasive sampling. The heterogeneity of these modalities prevents the simple aggregation of all positive findings as equivalent evidence of osseointegration.

## 4. Discussion

### 4.1. Interpretation of the Evidence

Across the 19 included human studies, the most consistent favorable signal was observed during early healing, particularly as a faster recovery or increase in RFA‐derived implant stability quotient (ISQ). Several studies reported higher early ISQ values or a more favorable stability trajectory at PRF‐treated sites [20, 22, 24, 30, 33, 37], whereas other trials found no significant benefit [29, 34, 36, 38]. In Kapoor et al. [20], the early advantage was no longer evident at 3 months. This pattern suggests that some PRF protocols may accelerate the transition from primary mechanical stability to secondary biological stability rather than increase the final extent of osseointegration. Evidence at 6–12 months was less consistent, follow‐up beyond 1 year was scarce, and no included study demonstrated a durable advantage in implant survival. Early ISQ or radiographic changes should therefore not be described as long‐term superiority.

PRF formulations were also interpreted separately. Solid L‐PRF was the most frequently studied and showed mixed effects across osteotomy, immediate‐implant, and socket applications. Evidence for A‐PRF was limited mainly to a socket‐healing comparison without an untreated control [32]. The i‐PRF/liquid‐PRF studies were few and clinically heterogeneous: Güvenç et al. [37] and Hasanzade et al. [24] reported favorable early stability, Mohye et al. [21] reported higher radiographic density when i‐PRF was combined with a xenograft, and De Oliveira Fernandes et al. [34] found no significant benefit from liquid‐PRF coating through 1 year. These interventions are not interchangeable, and a result obtained with one formulation or delivery method cannot be generalized to the entire PRF family. The study identified by PMID 28165420 provides in vitro mechanistic information but was appropriately excluded from the 19‐study clinical evidence set [43, 44].

### 4.2. Methodological and Protocol Considerations

Interpretation is further complicated using different outcome methods. RFA is noninvasive and useful for monitoring the early stability transition, but ISQ reflects the stiffness of the implant‐bone complex rather than direct bone‐to‐implant contact [39]. Insertion torque measures mechanical resistance at placement and is influenced by osteotomy preparation and cortical engagement; it is not interchangeable with RFA [40]. Periapical radiographs assess mesial and distal marginal bone but not facial or lingual surfaces, while CBCT provides three‐dimensional information, but its gray values are affected by the scanner and acquisition protocol and should not be treated as calibrated Hounsfield units without validation [41, 42]. Histology is biologically more specific but is rarely available in human studies and may sample adjacent bone rather than the functional implant interface. Thus, Kapoor et al.’s [20] transient RFA difference and Mohye et al.’s [21] density finding are not necessarily contradictory because they represent different constructs, time points, and cointerventions.

Preparation heterogeneity may also contribute to inconsistent findings. Reporting rpm and time alone is insufficient because the RCF varies with rotor radius and geometry. Reproducible protocols should specify the PRF subtype, blood volume, tube material and manufacturer, collection‐to‐spin interval, centrifuge model, rotor angle and radius, rpm, RCF at a defined point, duration, harvested fraction, and postcentrifugation handling [8, 13]. Horizontal centrifugation and C‐PRF may improve cellular distribution or growth‐factor delivery in laboratory studies, but these approaches were not adequately represented in the eligible implant trials and cannot yet be considered clinically superior [10–12]. Tube composition is likewise part of the intervention; reports of silica microparticles and acute cellular effects from some coated plastic tubes support the need for validated devices and transparent reporting, although they do not establish clinical harm [14, 15].

The included evidence was also heterogeneous in design, sample size, implant site and timing, defect morphology, implant system, loading protocol, comparator, cointervention, and follow‐up. Small samples, incomplete reporting of allocation concealment or assessor blinding, and uncertain handling of paired observations in some split‐mouth trials reduce confidence in both positive and neutral results. A formal risk‐of‐bias assessment was not undertaken because this JBI scoping review was designed to map the evidence rather than estimate a pooled treatment effect [19]. Nevertheless, the absence of formal appraisal limits the ability to rank individual studies; accordingly, the findings should be interpreted as evidence mapping rather than as a graded clinical recommendation.

### 4.3. Clinical Relevance and Patient Perspective

PRF is autologous and can be prepared chairside, but patient benefit was insufficiently evaluated. Only Lima et al. recorded postoperative pain using a visual analog scale and analgesic consumption; because this was an uncontrolled case series, the findings cannot establish that PRF reduced postoperative discomfort [27]. The remaining studies did not systematically assess pain or analgesic use, and none evaluated esthetic satisfaction, treatment acceptance, oral‐health‐related quality of life, or time to normal activity. Clinically recorded healing or peri‐implant indices should not be treated as substitutes for patient‐reported outcomes.

Clinical use also requires venipuncture, validated centrifuge and tubes, trained staff, strict timing, and additional chairside processing. No included study performed a formal cost‐effectiveness analysis; therefore, claims of low cost should account for equipment, consumables, staff time, failed preparations, and whether PRF reduces graft use, complications, healing time, or additional visits. At present, PRF may be considered an optional adjunct for a defined indication and a reproducible protocol, but it should not compensate for inadequate primary stability, poor implant positioning, uncontrolled infection, deficient tissue management, or inappropriate loading. Patients should be informed that the evidence is more suggestive of early healing than for long‐term implant success.

### 4.4. Limitations and Research Priorities

This review was restricted to three databases, English‐language publications, and studies published from 2015 to 2025. Metaanalysis was not performed because of the scoping objective and substantial clinical, protocol, and outcome heterogeneity. The predominance of small studies, short follow‐up, surrogate endpoints, incompletely reported preparation protocols, and combined interventions limits the causal interpretation. Patient‐reported and economic outcomes were largely absent, and no study established multiyear superiority in implant success or survival.

Future trials should compare clearly defined PRF formulations under otherwise comparable clinical conditions; report the complete tube, device, centrifugation, and handling protocol; use adequately powered randomized designs with appropriate analysis of split‐mouth clustering; and distinguish early biological outcomes from long‐term clinical performance. A core outcome set should combine standardized RFA and marginal bone assessment with implant success and survival, peri‐implant health, prosthetic complications, postoperative pain, analgesic consumption, satisfaction, oral‐health‐related quality of life, and cost‐effectiveness. These measures are necessary to determine whether an early surrogate improvement produces a meaningful and durable benefit for patients.

## 5. Conclusion

PRF and their derivatives show promising potential as autologous adjuncts to dental implant treatment, principally through a possible acceleration of early healing and secondary implant stability. The effect is not consistent across studies, preparation protocols, or outcome methods, and early ISQ or radiographic changes should not be equated with durable osseointegration. Current evidence does not establish long‐term superiority in marginal bone preservation, implant success, or implant survival, and it does not support designating i‐PRF or any other PRF formulation as universally reliable or superior. Well‐designed, adequately powered randomized trials using standardized, fully reported preparation protocols, complementary validated outcome measures, long‐term follow‐up, patient‐reported outcomes, and economic evaluation are required before routine clinical recommendations can be made.

## Author Contributions

**P. Gitanjali**: conceptualization, methodology, investigation, literature screening, study selection, data curation, formal analysis, writing – original draft preparation, writing – review and editing, visualization, project administration. **Neetha J. Shetty**: conceptualization, methodology, validation, formal analysis, supervision, writing – review and editing. **Amitha Basheer**: investigation, data curation, validation, writing – review and editing, assisted in literature screening, study selection, data extraction, organization of study characteristics. **Shruti Singh**: investigation, data curation, validation, writing – review and editing, assisted in literature screening, data extraction, verification of the included studies. **Nandini Nagpal**: methodology, validation, formal analysis, writing – review and editing, assisted in the synthesis and interpretation of the extracted evidence, critical revision of the manuscript. **Shreya Hegde**: data curation, validation, writing – review and editing, assisted in data verification, formatting of tables and references, final manuscript editing.

## Funding

This study did not receive any specific funding.

## Disclosure

All authors have read and approved the final version of the manuscript. Neetha J. Shetty, the corresponding author, had full access to all the data in this study and takes complete responsibility for the integrity of the data and the accuracy of the data analysis. No artificial intelligence instruments were employed for data extraction, analysis, interpretation, or manuscript composition.

## Ethics Statement

This scoping review synthesized data from previously published studies and did not involve the recruitment of human participants, collection of identifiable personal information, or use of human biological specimens. Therefore, institutional ethical approval and informed consent were not required.

## Conflicts of Interest

The authors declare no conflicts of interest.

## Data Availability

No new data were generated. All data were obtained from published sources available in public databases (PubMed/MEDLINE, Web of Science, and Embase) and via the articles cited. The authors confirm that the data supporting the findings of this study are available within the article.

## References

[1] Calciolari E. , Dourou M. , Akcali A. , and Donos N. , Differences Between First- and Second-Generation Autologous Platelet Concentrates, Periodontology 2000. (2025) 97, no. 1, 52–73, 10.1111/prd.12550. 38487938 PMC11808449

[2] Ivanovski S. , Lee R. S. B. , Fernandez-Medina T. , Pinto N. , Andrade C. , and Quirynen M. , Impact of Autologous Platelet Concentrates on the Osseointegration of Dental Implants, Periodontology 2000. (2025) 97, no. 1, 271–286, 10.1111/prd.12563. 38647020 PMC11808427

[3] Blanco J. , García A. , Hermida-Nogueira L. , and Castro A. B. , How to Explain the Beneficial Effects of Leukocyte- and Platelet-Rich Fibrin, Periodontology 2000. (2025) 97, no. 1, 74–94, 10.1111/prd.12570. 38923566 PMC11808445

[4] Choukroun J. , Diss A. , and Simonpieri A. , et al.Platelet-Rich Fibrin (PRF): A Second-Generation Platelet Concentrate. Part IV: Clinical Effects on Tissue Healing, Oral Surgery, Oral Medicine, Oral Pathology, Oral Radiology, and Endodontology. (2006) 101, no. 3, 299–303, 10.1016/j.tripleo.2005.07.012. 16504852

[5] Ghanaati S. , Booms P. , and Orlowska A. , et al.Advanced Platelet-Rich Fibrin: A New Concept for Cell-Based Tissue Engineering by Means of Inflammatory Cells, Journal of Oral Implantology. (2014) 40, no. 6, 679–689, 10.1563/aaid-joi-D-14-00138. 24945603

[6] Fujioka-Kobayashi M. , Miron R. J. , Hernandez M. , Kandalam U. , Zhang Y. , and Choukroun J. , Optimized Platelet-Rich Fibrin With the Low-Speed Concept: Growth Factor Release, Biocompatibility, and Cellular Response, Journal of Periodontology. (2017) 88, no. 1, 112–121, 10.1902/jop.2016.160443. 27587367

[7] Miron R. J. , Fujioka-Kobayashi M. , and Hernandez M. , et al.Injectable Platelet Rich Fibrin (i-PRF): Opportunities in Regenerative Dentistry?, Clinical Oral Investigations. (2017) 21, no. 8, 2619–2627, 10.1007/s00784-017-2063-9. 28154995

[8] Miron R. J. , Fujioka-Kobayashi M. , Sculean A. , and Zhang Y. , Optimization of Platelet-Rich Fibrin, Periodontology 2000. (2024) 94, no. 1, 79–91, 10.1111/prd.12521. 37681522

[9] Quirynen M. , Blanco J. , and Wang H. L. , et al.Instructions for the Use of L-PRF in Different Clinical Indications, Periodontology 2000. (2025) 97, no. 1, 420–432, 10.1111/prd.12564. 38803016 PMC11808411

[10] Miron R. J. , Chai J. , and Zhang P. , et al.A Novel Method for Harvesting Concentrated Platelet-Rich Fibrin (C-PRF) With a 10-Fold Increase in Platelet and Leukocyte Yields, Clinical Oral Investigations. (2020) 24, no. 8, 2819–2828, 10.1007/s00784-019-03147-w. 31788748

[11] Fujioka-Kobayashi M. , Katagiri H. , and Kono M. , et al.Improved Growth Factor Delivery and Cellular Activity Using Concentrated Platelet-Rich Fibrin (C-PRF) When Compared With Traditional Injectable (i-PRF) Protocols, Clinical Oral Investigations. (2020) 24, no. 12, 4373–4383, 10.1007/s00784-020-03303-7. 32382929

[12] Farshidfar N. , Apaza Alccayhuaman K. A. , and Estrin N. E. , et al.Advantages of Horizontal Centrifugation of Platelet-Rich Fibrin in Regenerative Medicine and Dentistry, Periodontology 2000. (2025) 99, no. 1, 216–267, 10.1111/prd.12625. 40130760 PMC13428090

[13] Salgado-Peralvo Á.O. , Kewalramani N. , and Pérez-Jardón A. , et al.Understanding Solid-Based Platelet-Rich Fibrin Matrices in Oral and Maxillofacial Surgery: An Integrative Review of the Critical Protocol Factors and Their Influence on the Final Product, Medicina. (2023) 59, no. 11, 10.3390/medicina59111903, 1903. 38003952 PMC10673335

[14] Tsujino T. , Takahashi A. , and Yamaguchi S. , et al.Evidence for Contamination of Silica Microparticles in Advanced Platelet-Rich Fibrin Matrices Prepared Using Silica-Coated Plastic Tubes, Biomedicines. (2019) 7, no. 2, 10.3390/biomedicines7020045. PMC6631693 31248187

[15] Masuki H. , Isobe K. , and Kawabata H. , et al.Acute Cytotoxic Effects of Silica Microparticles Used for Coating of Plastic Blood-Collection Tubes on Human Periosteal Cells, Odontology. (2020) 108, no. 4, 545–552, 10.1007/s10266-020-00486-z. 31997225 PMC7438384

[16] Guan S. , Xiao T. , and Bai J. , et al.Clinical Application of Platelet-Rich Fibrin to Enhance Dental Implant Stability: A Systematic Review and Meta-Analysis, Heliyon. (2023) 9, no. 2, 10.1016/j.heliyon.2023.e13196, e13196. 36785817 PMC9918761

[17] Lyris V. , Millen C. , and Besi E. , et al.Effect of Leukocyte and Platelet Rich Fibrin (L-PRF) on Stability of Dental Implants: A Systematic Review and Meta-Analysis, British Journal of Oral and Maxillofacial Surgery. (2021) 59, no. 10, 1130–1139, 10.1016/j.bjoms.2021.01.001. 34702597

[18] Tricco A. C. , Lillie E. , and Zarin W. , et al.PRISMA Extension for Scoping Reviews (PRISMA-ScR): Checklist and Explanation, Annals of Internal Medicine. (2018) 169, no. 7, 467–473, 10.7326/M18-0850. 30178033

[19] Peters M. D. J. , Marnie C. , and Tricco A. C. , et al.Updated Methodological Guidance for the Conduct of Scoping Reviews, JBI Evidence Synthesis. (2020) 18, no. 10, 2119–2126, 10.11124/JBIES-20-00167. 33038124

[20] Kapoor A. , Ali A. R. , Saini N. , Gautam K. , Goyal A. , and Prakash V. , Comparative Evaluation of Implant Stability With and Without Autologous Platelet-Rich Fibrin Prior to Prosthetic Loading—A Split-Mouth Randomized Clinical Trial, Journal of Indian Society of Periodontology. (2022) 26, no. 2, 137–142, 10.4103/jisp.jisp_74_21. 35321306 PMC8936022

[21] Mohye B. , Al Mofty M. S. , and Gamal A. Y. , The Effect of Using of i-PRF (Injectable Platelet Rich Fibrin) on Filling the Gap of Immediate Dental Implant in the Esthetic Zone: Randomized Clinical Trial, Journal of the International Academy of Periodontology. (2022) 24, no. 3, 273–282.

[22] Öncü E. and Alaaddinoğlu E. E. , The Effect of Platelet-Rich Fibrin on Implant Stability, The International Journal of Oral & Maxillofacial Implants. (2015) 30, no. 3, 578–582, 10.11607/jomi.3897. 26009908

[23] Ali A. R. , Kumar P. , Gautam K. , Vaish S. , Choudhary A. , and Fareetha Fowzana S. , Clinical and Radiographic Assessment of Effect of Platelet-Rich Fibrin on Dental Implant Osseointegration—A Split Mouth Randomized Clinical Trial, Journal of Prosthodontics-Implant Esthetic and Reconstructive Dentistry. (2025) 34, no. 5, 469–477, 10.1111/jopr.14004. 39763166

[24] Hasanzade T. , Ertugrul A. S. , and Yuzbasioglu Ertugrul B. , Effects of Injectable Platelet-Rich Fibrin on the Osseointegration of Dental Implants, Medical Science Monitor: International Medical Journal of Experimental and Clinical Research. (2025) 31, 10.12659/MSM.949298, e949298. 40974036 PMC12459210

[25] Awasthi N. , Sonkesriya S. , Samal S. K. , Reddy U. , Felmban M. H. M. , and Santosh S. H. , A Clinical Evaluation of Immediate Implant Placement With/Without PRF in Anterior Esthetic Zone, The Journal of Contemporary Dental Practice. (2025) 26, no. 7, 679–682, 10.5005/jp-journals-10024-3911. 41045162

[26] Sharma K. , Roy S. , and Kumari A. , et al.A Comparative Evaluation of Soft and Hard Tissue Changes Around Dental Implants Placed With and Without Platelet-Rich Fibrin, Cureus. (2023) 15, no. 3, 10.7759/cureus.36908, e36908. 37128513 PMC10148605

[27] Lima V. C. S. , Miguel M. M. V. , Ferraz L. D. F. F. , Filho A. B. M. , Jardini M. A. N. , and Santamaria M. P. , Use of Platelet-Rich Fibrin Membranes With Single Implant Placement for Peri-Implant Mucosal Thickness Augmentation: A Case Series Study, Clinical Advances in Periodontics. (2022) 12, no. 1, 17–20, 10.1002/cap.10143. 33340395

[28] Boora P. , Rathee M. , and Bhoria M. , Effect of Platelet Rich Fibrin on Peri-Implant Soft Tissue and Crestal Bone in One-Stage Implant Placement: A Randomized Controlled Trial, Journal of Clinical and Diagnostic Research. (2015) 9, no. 4, 10.7860/JCDR/2015/12636.5788. PMC4437152 26023636

[29] Naeimi Darestani M. , Asl Roosta H. , Mosaddad S. A. , and Yaghoubee S. , The Effect of Leukocyte- and Platelet-Rich Fibrin on the Bone Loss and Primary Stability of Implants Placed in Posterior Maxilla: A Randomized Clinical Trial, International Journal of Implant Dentistry. (2023) 9, no. 1, 10.1186/s40729-023-00487-x. PMC10412516 37555894

[30] Anapu M. P. , Atluri K. R. , and Chandra Tripuraneni S. , et al.Evaluation of Effect on Stability of Implants With and Without Platelet Rich Fibrin Using a Resonance Frequency Analyzer: An in-Vivo Study, Heliyon. (2024) 10, no. 7, 10.1016/j.heliyon.2024.e27971, e27971. 38623195 PMC11016576

[31] Tamilarasan M. , Nivetha R. , and Prabhahar C. S. , et al.Evaluation of Primary and Secondary Stability of Endosseous Dental Implants With and Without the Use of Platelet-Rich Fibrin: A Clinical Study, Cureus. (2024) 16, no. 6, 10.7759/cureus.62918, e62918. 39040770 PMC11262779

[32] Chary N. O. B. P. , Raju M. S. , Sajjan M. C. S. , Gottumukkala S. N. V. S. , and Manyam R. , Comparison of Quality of Bone and Insertion Torque Values of Early Implants Placed at 6 and 8 Weeks in Sockets Preserved With Advanced Platelet-Rich Fibrin: A Randomized Controlled Trial, The Journal of Indian Prosthodontic Society. (2021) 21, no. 4, 366–374, 10.4103/jips.jips_331_21. 34810364 PMC8617450

[33] Tabrizi R. , Arabion H. , and Karagah T. , Does Platelet-Rich Fibrin Increase the Stability of Implants in the Posterior of the Maxilla? A Split-Mouth Randomized Clinical Trial, International Journal of Oral and Maxillofacial Surgery. (2018) 47, no. 5, 672–675, 10.1016/j.ijom.2017.07.025. 29269149

[34] de Oliveira Fernandes G. , Santos N. , de Sousa M. , and Fernandes J. , Liquid Platelet-Rich Fibrin Coating Implant Surface to Enhance Osseointegration: A Double-Blinded, Randomized Split-Mouth Trial With 1-Year Follow-up, The International Journal of Oral & Maxillofacial Implants. (2022) 37, no. 1, 159–170, 10.11607/jomi.9107. 35235635

[35] Jalaluddin M. , Ramanna P. K. , and Jasthi V. C. , et al.Comparative Assessment of the Oral Implant Stability With/Without Platelet-Rich Fibrin: An in Vivo Study, The Journal of Contemporary Dental Practice. (2025) 26, no. 5, 500–503, 10.5005/jp-journals-10024-3801. 40960107

[36] Soni M. , Gugnani S. , Pandit N. , Bali D. , and Sharma M. , Efficacy of Leukocyte-Platelet-Rich Fibrin Membrane in Immediate Postextraction Implant Placement: A Randomized Controlled Trial, Journal of Indian Society of Periodontology. (2023) 27, no. 1, 63–69, 10.4103/jisp.jisp_219_21. 36873981 PMC9979809

[37] Güvenç S. , Durmuşlar M. C. , and Ballı U. , The Effects of Injectable Platelet-Rich Fibrin on Implant Stability, The International Journal of Oral & Maxillofacial Implants. (2022) 37, no. 6, 1145–1150, 10.11607/jomi.9629. 36450019

[38] Diana C. , Mohanty S. , Chaudhary Z. , Kumari S. , Dabas J. , and Bodh R. , Does Platelet-Rich Fibrin Have a Role in Osseointegration of Immediate Implants? A Randomized, Single-Blind, Controlled Clinical Trial, International Journal of Oral and Maxillofacial Surgery. (2018) 47, no. 9, 1178–1188, 10.1016/j.ijom.2018.01.001. 29402513

[39] Atsumi M. , Park S. H. , and Wang H. L. , Methods Used to Assess Implant Stability: Current Status, The International Journal of Oral & Maxillofacial Implants. (2007) 22, no. 5, 743–754. 17974108

[40] Lages F. S. , Douglas-de Oliveira D. W. , and Costa F. O. , Relationship Between Implant Stability Measurements Obtained by Insertion Torque and Resonance Frequency Analysis: A Systematic Review, Clinical Implant Dentistry and Related Research. (2018) 20, no. 1, 26–33, 10.1111/cid.12565. 29194944

[41] Pauwels R. , Jacobs R. , Singer S. R. , and Mupparapu M. , CBCT-Based Bone Quality Assessment: Are Hounsfield Units Applicable?, Dentomaxillofacial Radiology. (2015) 44, no. 1, 10.1259/dmfr.20140238, 20140238. 25315442 PMC4277442

[42] Eguren M. , Holguin A. , and Diaz K. , et al.Can Gray Values be Converted to Hounsfield Units? A Systematic Review, Dentomaxillofacial Radiology. (2022) 51, no. 1, 10.1259/dmfr.20210140, 20210140. 34148350 PMC8693322

[43] Wang X. , Zhang Y. , Choukroun J. , Ghanaati S. , and Miron R. J. , Behavior of Gingival Fibroblasts on Titanium Implant Surfaces in Combination With Either Injectable-PRF or PRP, International Journal of Molecular Sciences. (2017) 18, no. 2, 10.3390/ijms18020331. PMC5343867 28165420

[44] Albrektsson T. , Brånemark P. I. , Hansson H. A. , and Lindström J. , Osseointegrated Titanium Implants: Requirements for Ensuring a Long-Lasting, Direct Bone-to-Implant Anchorage in Man, Acta Orthopaedica Scandinavica. (1981) 52, no. 2, 155–170, 10.3109/17453678108991776. 7246093

